# Improving air quality needs to be a policy priority for governments globally

**DOI:** 10.1371/journal.pmed.1003041

**Published:** 2020-02-06

**Authors:** Aziz Sheikh

**Affiliations:** Usher Institute, The University of Edinburgh, Edinburgh, United Kingdom

## Abstract

In this Perspective, Aziz Sheikh discusses the importance of research to understand the impact of air pollution on human health, commenting on a study by Yaohua Tian and colleagues that examined associations between ambient air quality and risk of hospitalization for pneumonia in adults in China

There is increasing international interest in understanding the impact of ambient air pollution on human health. The existing body of evidence has demonstrated potentially important associations between air quality and the risks of cardiovascular [1,2], neurovascular [1], and chronic respiratory disorders [2,3]. Most of this evidence comes from high-income countries and has, in many such countries, triggered policy initiatives aimed at improving air quality. These include, for example, the creation of low-emission zones (LEZs), which have led to restrictions on the use of the heaviest polluting vehicles within urban areas [4]. The evidence of the impact of air pollution on respiratory tract infections is, by contrast, sparse and inconclusive, this reflecting the still-emerging understanding of the myriad of ways in which atmospheric pollutants can impact human health and the considerable challenges in mounting the large-scale, time-series, and experimental studies needed to generate a robust evidence base [5]. The study by Yaohua Tian and colleagues published in *PLOS Medicine* studied the impact of ambient air quality on the risk of hospitalizations for pneumonia in Chinese adults, and it is welcome on account of the experimental design employed, its size, and the fact that this is one of the first such evaluations to have been undertaken in low- and middle-income countries (LMICs) [6].

Using data on ambient particulate measure (PM) concentrations, measured through a national network of air quality monitors, the authors were able to investigate the association between short-term PM concentration and risk of hospital admission for pneumonia in adults. These hospitalization data were obtained from the national Urban Employee Basic Medical Insurance (UEBMI), one of 3 national health insurance schemes, which covers over 280 million adults from across 184 Chinese cities. In total, the authors were able to study 4.2 million pneumonia admissions over a 3-year period (2014 to 2017). They found that short-term (3-day) elevations in PM were associated with increased risk of pneumonia admissions in adults. These associations were seen with increases of both PM_2.5_ and PM_10_ concentrations.

Key strengths of this study were its scale (both in terms of its national-, regional-, and city-level coverage and the numbers of admissions being studied), the quality of the data on PM concentration (overall and separately for PM_10_ and PM_2.5_), and the use of the hard outcome measure of International Classification of Diseases (ICD)-coded hospital admission for pneumonia. Further strengths include the time-series design and the fact that these data come from Asia, where ambient PM exposure is typically much higher than in most high-income country settings [7]. Importantly, the authors were able to demonstrate how changes in temperature and humidity modified these associations.

There are, however, limitations of the study that need to be considered. These include, most fundamentally, the ecological study design employed, which limits the opportunity to adjust for potential confounders between air pollution and risk of pneumonia admission—for example, smoking status [8] and passive smoking [9]. Other limitations include only studying hospital admissions for pneumonia; we thus have no understanding of what the impact was, if any, on cases of pneumonia that were not severe enough to warrant hospital admission or, conversely, more severe episodes that led to death from pneumonia outside of a hospital setting. The unavailability of ICD-coded hospital admission data from major cities such as Beijing and Shanghai is another potential cause for concern, as this may have resulted in biased estimates. From a policy perspective, in addition to attending to the aforementioned concerns, it would have been helpful to broaden the range of outcomes being studied to a wider array of respiratory and nonrespiratory conditions—for instance, asthma, chronic obstructive pulmonary disease (COPD), ischemic heart disease, and stroke. This is because the availability of such evidence is likely to help those in a position to promote policy initiatives to make the case for investments in improving air quality and health while also considering the potential impact of such interventions on transport and trade [10].

Going forward, there is then a need to extend this work to other respiratory and other relevant nonrespiratory conditions to obtain an overall population-level assessment of the impact of air quality on human health in China and other countries. Such evidence will be helpful in making the case to invest in interventions to improve air quality, which, ideally, should then be independently evaluated [11]. In high-income countries, these interventions mainly center on measures to curb vehicle emissions; in LMIC environments, these interventions are also likely to include measures to reduce industrial, agricultural (from the burning of crops), and domestic (from biomass fuel [12]) emissions.

As Tian and colleagues’ study and the growing body of international evidence now demonstrates, ambient air pollution is likely to pose one of the greatest threats to human health [13], and through its impact on climate change, it also represents perhaps one of the greatest threats to planetary health. Although there is still much to be done to better understand the nature of these relationships, given the potential catastrophic consequences for our individual and collective health, it is imperative that governments globally continue to push improvement of air quality to the very top of their policy priorities.

## Abbreviations

COPD: chronic obstructive pulmonary disease
ICD: International Classification of Diseases
LEZ: low-emission zone
LMIC: low- and middle-income countries
PM: particulate measure
UEBMI: Urban Employee Basic Medical Insurance
